# Factors associated with not smoking among Aboriginal and Torres Strait Islander adolescents and young people: Analysis of data from the 2014 to 15 National Aboriginal and Torres Strait Islander Social Survey

**DOI:** 10.1002/hpja.942

**Published:** 2024-12-26

**Authors:** Emilie Cameron, Megan Freund, Sandra Eades, Nicole Turner, Robert Davis, Christina Heris, Jennifer Rumbel, Matthew Clapham, Jamie Bryant

**Affiliations:** ^1^ Health Behaviour Research Collaborative, School of Medicine and Public Health, Faculty of Health and Medicine University of Newcastle Callaghan New South Wales Australia; ^2^ Equity in Health and Wellbeing Program, Hunter Medical Research Institute New Lambton Heights New South Wales Australia; ^3^ Centre for Epidemiology and Biostatistics, School of Population and Global Health University of Melbourne Carlton Victoria Australia; ^4^ NSW Rural Doctors Network St Leonards New South Wales Australia; ^5^ National Centre for Aboriginal and Torres Strait Islander Wellbeing Research, National Centre for Epidemiology and Population Health, Australian National University College of Health and Medicine Canberra Australian Capital Territory Australia; ^6^ Systems Neuroscience Group, School of Psychological Sciences, Hunter Medical Research Institute New Lambton Heights New South Wales Australia; ^7^ Wollotuka Institute University of Newcastle Callaghan New South Wales Australia; ^8^ Clinical Research Design, Information Technology and Statistical Support, Hunter Medical Research Institute New Lambton Heights New South Wales Australia

**Keywords:** adolescent behaviour, Australian Aboriginal and Torres Strait Islander peoples, Indigenous peoples, smoking prevention, tobacco smoking

## Abstract

**Issue Addressed:**

Smoking rates have been steadily declining among Aboriginal and Torres Strait Islander people. Examining the factors associated with not smoking in young people is crucial for understanding the motivations and influences that lead individuals to adopt healthy behaviours.

**Methods:**

Secondary analysis was undertaken of data collected as part of the National Aboriginal and Torres Strait Islander Social Survey (NATSISS) 2014–15 (*n* = 1456). Factors associated with not smoking were explored with three multivariate regressions: (1) socio‐demographic characteristics; (2) health, social and emotional factors; and (3) cultural identity characteristics.

**Results:**

Overall, 66% of Aboriginal and Torres Strait Islander young people aged 15–24 years did not smoke. Factors associated with not smoking included being younger, female, and engaged in study or employment. Those who lived with no smoking in the house, had lower illicit drug and alcohol use, and participated in Aboriginal or Torres Strait Islander sports carnivals were also more likely to be non‐smokers.

**Conclusions:**

Study findings reinforce the influence of social determinants on smoking behaviour. Efforts to reduce smoking among Aboriginal and Torres Strait Islander young people should focus on removing barriers to education and employment, promoting positive peer and family influences within households, taking a multi‐drug approach to cessation, and considering cultural identity and its role in promoting healthy lifestyles.

**So What?:**

Understanding the protective factors associated with not smoking in young Aboriginal and Torres Strait Islander people will help with developing effective policies and initiatives to improve health outcomes.

## 1 INTRODUCTION

Smoking continues to be the biggest contributor to preventable disease globally. Each year, over 8 million people die from tobacco‐related causes.^1^ The process of colonisation, which in Australia included the forceful takeover of land, disruption of cultural practices, and systemic marginalisation of Indigenous communities,^2^ had a substantial and enduring impact on the health and wellbeing of Aboriginal and Torres Strait Islander people.^3^ In Australia, approximately half of all deaths among Aboriginal and Torres Strait Islander people aged 45 years and older are attributable to tobacco smoking.^4^ Demonstrating resilience in the face of inequality and disruptions to culture, rates of tobacco use have been steadily declining for Aboriginal and Torres Strait Islander people over the past 20 years. In 2021, 37% of Aboriginal and Torres Strait Islander people aged 15 years and older were daily smokers, compared to 50% in 2002.^5^

As for most adults who smoke, adult First Nations people most often commence smoking during adolescence.^6^, ^7^ Adolescence, in general, is a challenging time of transition. Adolescents are particularly susceptible to nicotine addiction and the tactics used by tobacco companies to encourage smoking initiation and establish smoking as a lifelong habit.^8^ However, there is evidence to indicate that smoking among Aboriginal and Torres Strait Islander people in adolescence is decreasing. Between 2005 and 2017, the proportion of Aboriginal and Torres Strait Islander secondary students who had never smoked increased from 49% to 70%, and the intensity of smoking among current smokers reduced.^9^ In addition, the proportion of smokers aged 18–24 who started smoking before the age of 18 declined between 2004 and 2013 from 84% to 76%, suggesting that the age of smoking initiation is increasing.^10^

To inform prevention activities aimed at reducing the smoking‐related burden among Aboriginal and Torres Strait Islander young people, it is important to understand the factors that influence smoking. Research that has examined smoking among adolescents globally has found a range of behavioural, psychosocial, and environmental factors influencing smoking behaviour. This includes socioeconomic status, academic performance, family and peer influence, rebelliousness, thrill seeking, risk‐taking behaviours and the prevalence of smoking in the community.^11^, ^12^ For First Nations people, these factors can be impacted by extra challenges, including racism and intergenerational trauma.^11^, ^13^, ^14^ To date, only limited research has examined the protective and risk factors for Aboriginal and Torres Strait Islander young people being smoke‐free. In a small survey of 106 adolescents from the same geographic area, participants who never smoked regularly were more likely to have good mental health, a stable home environment, less risk behaviours and no justice interactions.^15^ In yarning circles and focus groups conducted with young Aboriginal and Torres Strait Islander people, good mental and physical health, reduced stress and boredom, having less exposure to smoking by peers and in the home, and strong connections to family, community and culture were identified as factors promoting non‐smoking.^7^, ^16^, ^17^

Culture is of critical importance for Aboriginal and Torres Strait Islander people, serving as the cornerstone of their connection to ancestry, land, and community cohesion. It provides resilience in the face of historical traumas, empowers self‐expression, and fosters better health outcomes.^11^, ^18^, ^19^, ^20^ There is increasing recognition of the positive health benefits that identifying with culture can have.^19^, ^21^ Using yarning groups (a culturally safe relational research method grounded in Indigenous knowledges^22^), Tootell et al.^21^ identified the importance of knowing one's own mob (a term used to describe a group of Aboriginal and Torres Strait Islander people connected through a particular place or country), and connecting with both country and community more broadly for maintaining a strong cultural identity. Among Aboriginal and Torres Strait young people, factors such as cultural knowledge, community support, colonisation and agency have been identified as influencing perceptions of connectedness, and belonging.^23^ For a proportion of Aboriginal and Torres Strait islanders, these connections are disrupted by historical and contemporary child removal practices. No previous studies have reported on the influence of cultural factors on Aboriginal and Torres Strait Islander young people being smoke‐free.^11^

The aim of the current study was to examine the socio‐demographic, health and wellbeing, and cultural identity factors associated with not smoking among Aboriginal and Torres Strait Islander young people aged 15–24 years, using data collected as part of the National Aboriginal and Torres Strait Islander Social Survey (NATSISS) 2014/15.

## 2 METHODS

### 2.1 Aboriginal and Torres Strait Islander governance

An Aboriginal and Torres Strait Islander Reference Group was established to ensure appropriate Aboriginal overall oversight of the research process and interpretation of data. The Reference Group was made up of five Aboriginal members (including co‐authors SE, NT, RD and JR) with skills and expertise in Aboriginal health, health policy, evaluation, and the delivery of Aboriginal health care. The Reference Group provided advice, guidance and direction about the design, implementation, interpretation and dissemination of study findings. In particular, the Reference Group advised researchers about appropriate protocols and cultural sensitivities to enhance the relevance of the research project, as well as the impact of the Research Project on improving the health and wellbeing of Aboriginal and Torres Strait Islander peoples.

### 2.2 Study design

This is a secondary quantitative analysis of data from the 2014/2015 NATSISS. The NATSISS is a six‐yearly survey undertaken by the Australian Bureau of Statistics and obtains multidimensional self‐report social data from Aboriginal and Torres Strait Islander people living in private dwellings across Australia.^24^ In this study, we used multivariate regression analysis to examine the socio‐demographic, health and wellbeing, and cultural identity factors associated with not smoking.

### 2.3 Ethics approval

Ethical approval for the study was provided by the Aboriginal Health and Medical Research Council of NSW Ethics Committee (protocol number 1600/19) and registered with the University of Newcastle Human Research Ethics Committee (H‐2019‐0440). The research was conducted in line with the National Health and Medical Research Council's guidelines for ethical conduct in Aboriginal and Torres Strait Islander health research,^25^ the Aboriginal Health and Medical Research Council's ethical guidelines: key principles^26^ and the CONSIDER statement.^27^

### 2.4 Study sample

Data collection for the 2014–15 NATSISS was conducted from September 2014 to June 2015 in remote and non‐remote areas in all states and territories of Australia, including discrete Aboriginal and Torres Strait Islander communities. Interviews were conducted face‐to‐face by trained interviewers from the Australian Bureau of Statistics using a computer‐assisted interviewing questionnaire. Interviewers visited households in selected areas and conducted a screening process to identify those where one or more household members identified as being of Aboriginal or Torres Strait Islander. Data about the general characteristics of the household, including demographics details of household members, were collected from a household spokesperson aged 18 years or over. Based on this demographic information, up to two Aboriginal or Torres Strait Islander people aged 15 years and over living in the household were randomly selected for participation. Parent or guardian consent was required for the participation of adolescents aged 15–17 years, and interviews could be conducted in the presence of another household member if requested. Proxy interview was used to collect information only from those over 15 who did not have sufficient English skills, or who may have had an illness or injury that prevented a personal interview.^24^ Complete data were collected from 11 178 Aboriginal and Torres Strait Islander people from 6611 households. For this study, all Aboriginal and Torres Strait Islander adolescents and young people aged 15–24 years who participated in the NATSISS 2014–15 were eligible for inclusion in the analysis.^24^ Data were securely provided to the researchers for secondary analysis through the Australian Bureau of Statistic's Data Laboratory.

### 2.5 Outcome measures

#### 2.5.1 Smoking status

Individuals were defined as current non‐smokers if they reported being a never smoker or an ex‐smoker at the time of the survey completion. Smokers included those who reported daily or occasional (at least once a week, or less than weekly) smoking.

### 2.6 Explanatory variables

A detailed explanation of each variable is provided in Data S1.

#### 2.6.1 Socio‐demographic characteristics

Participants were asked their age in years, sex (male/female), whether they were currently studying or employed (not employed or studying, studying, employed, both studying and employed) and their state or territory of residence. Geographical remoteness (2011 Accessibility/Remoteness Index of Australia Plus (ARIA+))^28^ classified as remote or non‐remote and the degree of socioeconomic disadvantage (2011 Socio‐Economic Indexes for Areas (SEIFA))^29^ sorted into quintiles were provided based on home postcode.

#### 2.6.2 Health, social and emotional wellbeing factors

Participants were asked about their self‐assessed health status (excellent, very good, good, fair or poor), whether anyone ever smokes inside the house (yes, no), single‐occasion alcohol risk (yes, no), lifetime alcohol risk (yes, no), total number of other illicit substances used (chosen from a list then summed), psychological distress measured by the Kessler‐5 (K‐5) scale (low, moderate, high, very high),^30^ whether told by a doctor or nurse that have a mental health condition including depression or feeling depressed, anxiety or feeling anxious or nervous, or behavioural or emotional problems (yes, no), and overall life satisfaction (rating on a 10‐point scale). Removal from family (yes, no, not answered) was explored through two questions that asked about the removal of self and the removal of relatives by welfare organisations or government, and/or being taken away to a mission.

#### 2.6.3 Cultural identity

Participants were asked if they speak any Aboriginal or Torres Strait Islander language (yes, some words, no), whether they currently live in homeland or traditional country (yes, no, don't recognise homelands), whether they identify with a tribal group, language group, clan, mission or other Aboriginal or Torres Strait Islander regional group (yes, no), and whether they had participated in any of the nine presented cultural activities, ceremonies, and traditional activities in the last 12 months (yes, no; see Table 1 for the list of activities).

### 2.7 Analysis

Statistical analysis for this secondary analysis of data from the 2014/2015 NATSISS was programmed using R version 4.1.1.^31^ Weighted population frequencies were calculated from the sample frequencies using a weighting scheme based on the total population of Aboriginal and Torres Strait Islander people who are residents of private dwellings. Further details regarding the survey methodology are available in the NATSISS 2014–15 Users' Guide.^24^ Population‐weighted proportions are presented by smoking status. Standard errors (SEs) were calculated with 250 replicate weights using the Jackknife method. Due to low numbers responding, ‘not stated’ and the need to maintain confidentiality, the response options ‘no’ and ‘not stated’ were combined for the variables smoking inside the house, lifetime alcohol risk and single‐occasion alcohol risk. The response options ‘low’ and ‘unable to determine’ were combined for K‐5. For other variables, the categories ‘Not stated’ or ‘Unable to determine’ were set to missing in the regression analysis. In all regressions, listwise deletion was used to handle missing values.

Factors associated with not smoking were explored with three multivariate regressions: Regression 1 included the socio‐demographic characteristics; Regression 2 included the health, social and emotional wellbeing characteristics; and Regression 3 included the cultural identity characteristics. Socio‐demographic characteristics with *p*‐values less than 0.1 in the crude models (age, sex, work or study, remoteness areas and socioeconomic status) were considered potential confounders and adjusted for in the subsequent regressions. All regressions used the estimated population frequencies. Risk ratios (RR) were estimated by marginal standardisation from logistic regressions. Crude and adjusted regression estimates are provided for the main variables. 95% confidence intervals (CI) were calculated with 250 replicate weights using the Jackknife method to account for the sampling design.

## 3 RESULTS

### 3.1 Sample description

Data for 1456 young people aged 15–24 across 1359 households were included in this analysis. This was weighted for the population size of 137 600. The population‐weighted characteristics of the survey sample are provided in Table 1. Roughly half were male (50.1%), and two‐thirds were aged over 18 years (66% 19–24 years). Most were either studying or employed (67.5%), resided in non‐remote parts of Australia (80.8%), had not been removed from family (92%), and had participated in an Aboriginal or Torres Strait Islander cultural or traditional activity in the last 12 months (65%). Overall, 66% did not smoke. This included 15% who were ex‐smokers and 85% who were never smokers. Among the 34% of the sample that smoked, 90% smoked daily and 10% were occasional smokers.

**TABLE 1:** Population‐weighted sample characteristics by smoking status (*N* = 137 600).

Variable	Category	Total % (SE)	Non‐smokers % (SE)
Smoking	Yes	34% (2.34)	–
	No	66% (2.34)	–
Socio‐demographic variables			
Age	15–16	20% (1.07)	81% (4.30)
	17–18	24% (1.42)	78% (3.89)
	19–20	22% (1.37)	57% (4.43)
	21–22	18% (1.31)	55% (5.43)
	23–24	16% (1.31)	55% (5.21)
Sex	Male	50% (<0.01)	62% (3.22)
	Female	50% (<0.01)	71% (2.63)
State or Territory	New South Wales	32% (0.05)	71% (4.21)
	Victoria	7.6% (0.12)	65% (3.86)
	Queensland	29% (0.03)	67% (4.87)
	South Australia	5.5% (0.17)	68% (6.19)
	Western Australia	13% (0.17)	63% (5.05)
	Tasmania	3.8% (0.13)	63% (3.95)
	Northern Territory	9.3% (0.17)	55% (4.33)
	Australian Capital Territory	1% (0.06)	67% (5.88)
Work or Study	Not employed or studying	33% (2.03)	45% (4.45)
	Employed	23% (1.53)	64% (4.46)
	Studying	26% (1.66)	79% (3.41)
	Both Studying and employed	19% (2.06)	88% (2.95)
Geographical remoteness (ARIA+)	Non‐remote	81% (<0.01)	70% (2.67)
	Remote	19% (<0.01)	51% (3.75)
Socioeconomic status (SEIFA)	Quintile 1 (most disadvantage)	51% (3.23)	59% (3.34)
	Quintile 2	21% (2.45)	75% (4.28)
	Quintile 3	13% (1.95)	68% (5.74)
	Quintile 4–5 (least disadvantage)	15% (2.36)	78% (5.69)
Health, social and emotional wellbeing characteristics			
Self‐assessed health status	Excellent	23% (1.84)	72% (4.45)
	Very good	31% (2.09)	71% (3.55)
	Good	33% (1.93)	62% (3.52)
	Fair	11% (1.17)	53% (5.60)
	Poor	2.2% (0.51)	58% (12.0)
Smoking inside house	Yes	15% (1.68)	43% (6.13)
	No	85% (1.68)	71% (2.32)
Lifetime (long‐term) alcohol risk	Yes	11% (1.29)	33% (6.37)
	No	90% (1.29)	70% (2.28)
Single occasion (short‐term) alcohol risk	Yes	26% (1.66)	47% (4.50)
	No	74% (1.66)	73% (2.39)
Number of different types of substances used in last 12 months	0	70% (2.10)	75% (2.44)
	1	21% (1.90)	53% (5.10)
	2	4.1% (0.71)	33% (7.86)
	3	1.9% (0.65)	25% (13.0)
	4+	2.6% (0.56)	28% (9.76)
Psychological distress (K5)	Low (5–7)	36% (2.20)	69% (3.79)
	Moderate (8–11)	32% (2.18)	69% (2.83)
	High (12–14)	16% (1.44)	64% (4.07)
	Very High (15–25)	17% (1.55)	58% (5.26)
Any mental health condition	Yes	22% (1.71)	65% (4.39)
	No	78% (1.71)	67% (2.53)
Ever removed from natural family	Yes	6.7% (1.03)	52% (8.72)
	No	92% (1.08)	67% (2.34)
	Not answered	1.2% (0.32)	52% (14.2)
Relatives ever removed from natural family	Yes	33% (2.33)	63% (4.16)
	No	54% (2.27)	68% (3.01)
	Not answered	14% (1.38)	65% (4.94)
Overall life satisfaction score	0–4 low satisfaction	5.9% (0.84)	51% (8.14)
	5–7	38% (2.10)	62% (3.50)
	8–10 high satisfaction	56% (2.07)	71% (2.92)
Cultural identity characteristics			
Speak any Aboriginal or Torres Strait Islander language	Yes	14% (1.21)	55% (5.10)
	Yes‐some words only	18% (1.58)	62% (5.01)
	No	68% (1.93)	70% (2.94)
Currently lives on homelands / traditional country	Yes	21% (1.67)	55% (4.58)
	No	42% (1.98)	67% (3.45)
	Does not recognise homelands	37% (2.08)	72% (3.51)
Identifies with clan, tribal or language group	Yes	52% (2.03)	64% (2.84)
	No	48% (2.03)	69% (3.31)
Participated in Aboriginal or Torres Strait Islander cultural events or traditional activities in last 12 months	Ceremonies	13% (1.36)	67% (4.71)
	Funerals / sorry business	28% (1.85)	61% (3.95)
	NAIDOC week activities	31% (2.11)	70% (4.16)
	Sports carnivals (excluding NAIDOC week activities)	22% (1.76)	71% (3.76)
	Festivals or carnivals involving arts, craft, music or dance (excluding NAIDOC week activities)	20% (1.74)	63% (5.02)
	Involved with Aboriginal and / or Torres Strait Islander organisations	13% (1.38)	75% (4.83)
	Fished/Hunted/Gathered wild plants/berries	56% (2.12)	61% (3.06)
	Made Aboriginal or Torres Strait Islander arts or crafts/Performed music, dance or theatre	21% (1.85)	75% (3.73)
	Written or told any Aboriginal and / or Torres Strait Islander stories	7.5% (1.10)	72% (6.55)
	None of the above	35% (2.05)	70% (3.31)

### 3.2 Socio‐demographic characteristics associated with not smoking

When accounting for other factors, those who were over 19 were significantly less likely to not smoke than those under the age of 16 (81% non‐smoking for ages 15–16 years compared to 57% for ages 19–20 years; RR = 0.74; 95% CI = 0.59–0.92) and females were significantly more likely to not smoke compared to males (71% vs. 62%; RR = 1.13; 95% CI = 1.02–1.25). The prevalence of not smoking was significantly higher among those who were currently studying (79%; RR = 1.46; 95% CI = 1.15–1.85), employed (64%; RR = 1.40; 95% CI = 1.14–1.72), or both (89%; RR = 1.70; 95% CI = 1.36–2.13), compared to those who were not studying or employed (45%). Those living in non‐remote areas were more likely to not smoke compared to those in remote areas in crude models, however, this was not significant when adjusting for other factors. Similarly, those in quintiles indicating least socioeconomic disadvantage were more likely to not smoke than those with most disadvantage in crude models, though this was not significant in the adjusted model (see Table 2).

**TABLE 2:** Socio‐demographic characteristics associated with not smoking among Aboriginal and Torres Strait Islander young people aged 15–24 years.

Variable	Category	Crude		Adjusted	
		RR (95% CI)	*p*‐value	RR (95% CI)	*p*‐value
Age	15–16	Reference	**<0.001**	Reference	**0.005**
	17–18	0.97 (0.83–1.12)		1.01 (0.84–1.22)	
	19–20	0.71 (0.58–0.86)		0.74 (0.59–0.92)	
	21–22	0.68 (0.55–0.85)		0.74 (0.59–0.92)	
	23–24	0.68 (0.56–0.83)		0.79 (0.63–0.98)	
Sex	Male	Reference	**0.017**	Reference	**0.016**
	Female	1.14 (1.02–1.27)		1.13 (1.02–1.25)	
State or Territory	New South Wales	Reference	0.246	Reference	0.906
	Victoria	0.92 (0.78–1.09)		0.93 (0.79–1.0)	
	Queensland	0.95 (0.8–1.12)		1.03 (0.87–1.21)	
	South Australia	0.96 (0.78–1.18)		1.02 (0.84–1.23)	
	Western Australia	0.89 (0.73–1.07)		1.04 (0.87–1.25)	
	Tasmania	0.89 (0.76–1.06)		0.93 (0.78–1.11)	
	Northern Territory	0.77 (0.63–0.95)		1.05 (0.87–1.26)	
	Australian Capital Territory	0.94 (0.77–1.16)		0.90 (0.71–1.15)	
Work or Study	None	Reference	**<0.001**	Reference	**<0.001**
	Employed	1.43 (1.13–1.79)		1.40 (1.14–1.72)	
	Studying	1.76 (1.42–2.16)		1.46 (1.15–1.85)	
	Both	1.95 (1.58–2.41)		1.70 (1.36–2.13)	
Remoteness Areas (ARIA+)	Remote	Reference	**<0.001**	Reference	0.061
	Non‐remote	1.37 (1.17–1.61)		1.16 (0.98–1.38)	
Socioeconomic status (SEIFA)	Quintile 1 (most disadvantage)	Reference	**0.004**	Reference	0.151
	Quintile 2	1.28 (1.1–1.5)		1.17 (1.01–1.36)	
	Quintile 3	1.17 (0.96–1.41)		1.03 (0.85–1.24)	
	Quintile 4–5 (least disadvantage)	1.33 (1.1–1.61)		1.17 (0.97–1.40)	

*Note*: Values significant at the 5% level are indicated in bold.

### 3.3 Health, social and emotional characteristics associated with not smoking

Accounting for other factors, a higher prevalence of not smoking was found among those who lived in a household where there was no smoking inside the dwelling compared to those who lived in a household where smoking occurred inside the dwelling (71% vs. 43%; RR = 1.42; 95% CI = 1.05–1.43), those who did not use other substances (such as illicit drugs or drugs for non‐medical purposes) compared to those who did (75% vs. 53%; RR = 1.85; 95% CI = 1.37–2.48), and those who had no single‐occasion alcohol consumption risk compared to those who did (74% vs. 47%; RR = 1.23; 95% CI = 1.05–1.43). There was no significant association between not smoking and having a mental health condition, level of psychological distress or being removed from natural family (see Table 3).

**TABLE 3:** Health, social and emotional wellbeing characteristics associated with associated with not smoking among Aboriginal and Torres Strait Islander young people aged 15–24 years.

Variable		Crude		Adjusted^a^	
	Category	RR (95% CI)	*p*‐value	RR (95% CI)	*p*‐value
Self‐assessed health	Poor	Reference	**0.026**	Reference	0.928
	Fair	0.92 (0.58–1.46)		1.08 (0.80–1.47)	
	Good	1.08 (0.72–1.61)		1.08 (0.84–1.39)	
	Very good	1.23 (0.81–1.85)		1.12 (0.86–1.47)	
	Excellent	1.24 (0.81–1.91)		1.11 (0.84–1.47)	
Smoking inside dwelling	Yes	Reference	**<0.001**	Reference	**<0.001**
	No	1.65 (1.25–2.19)		1.42 (1.13–1.77)	
Alcohol consumption‐ Single occasion risk	Yes	Reference	**<0.001**	Reference	**0.004**
	No	1.56 (1.29–1.88)		1.23 (1.05–1.43)	
Alcohol consumption‐ Lifetime risk	Yes	Reference	**<0.001**	Reference	0.179
	No	2.13 (1.45–3.12)		1.16 (0.91–1.49)	
Other substance use	2 or more	Reference	**<0.001**	Reference	**<0.001**
	1	1.79 (1.22–2.63)		1.45 (1.08–1.94)	
	0	2.52 (1.76–3.59)		1.85 (1.37–2.48)	
Psychological stress (K‐5)	Very high	Reference	0.212	Reference	0.924
	High	1.1 (0.9–1.35)		1.01 (0.85–1.19)	
	Moderate	1.18 (0.98–1.42)		1.01 (0.87–1.16)	
	Low	1.17 (0.87–1.41)		0.97 (0.83–1.13)	
Self‐report any mental health condition	Yes	Reference	0.628	Reference	0.895
	No	1.03 (0.9–1.19)		0.99 (0.87–1.13) 1.01 (0.88–1.15)	
Removal from natural family	Yes	Reference	0.088	Reference	0.232
	No	1.29 (0.93–1.79)		1.21 (0.90–1.62)	
	Not answered	1.01 (0.54–1.88)		1.31 (0.88–1.95)	
Relatives removed from natural family	Yes	Reference	0.453	Reference	0.209
	No	1.09 (0.93–1.27)		1.08 (0.97–1.22)	
	Not answered	1.04 (0.86–1.26)		0.97 (0.82–1.15)	
Overall life satisfaction	0–4 low satisfaction	Reference	0.018	Reference	0.673
	5–7	1.22 (0.87–1.69)		1.01 (0.79–1.30)	
	8–10 high satisfaction	1.38 (1.01–1.9)		1.07 (0.83–1.36)	

*Note*: Values significant at the 5% level are indicated in bold.

^a^ Adjusted for age, sex, work or study, remoteness areas, socioeconomic status, and all covariates presented in the table.

### 3.4 Cultural identity characteristics associated with not smoking

In the multivariate regression, there was a higher prevalence of not smoking in those who had attended Aboriginal or Torres Strait Islander sports carnivals, excluding National Aboriginal and Islander Day Observance Committee (NAIDOC) week activities, compared to those who had not (71% vs. 65%; RR = 1.16; 95% CI = 1.02–1.31). Conversely, those who attended Aboriginal or Torres Strait Islander Festivals or carnivals involving arts, craft, music or dance (excluding NAIDOC week activities) were less likely to not smoke than those who did not attend (63% vs. 67%; RR = 0.83; 95% CI = 0.69–1.00). There was no significant association between not smoking and participation in other Aboriginal or Torres strait Islander cultural events or traditional activities or ceremonies, living on homelands or traditional country, identifying with clan, tribal or language group, or speaking an Indigenous language (see Table 4).

**TABLE 4:** Cultural identity characteristics associated with associated with not smoking among Aboriginal and Torres Strait Islander young people aged 15–24 years.

			Crude		Adjusted^a^	
Variable		Category	RR (95% CI)	*p*‐value	RR (95% CI)	*p*‐value
Speak any Aboriginal or Torres Strait Islander language		No	Reference	**0.020**	Reference	0.330
		Yes‐some words only	0.88 (0.74–1.05)		0.89 (0.74–1.07)	
		Yes	0.78 (0.64–0.95)		0.96 (0.80–1.15)	
Currently live in homeland or traditional country		No	Reference	**0.013**	Reference	0.596
		Yes	0.82 (0.68–0.98)		0.93 (0.80–1.08)	
		Don't recognise an area as homeland or traditional country	1.07 (0.93–1.22)		1.01 (0.86–1.18)	
Identify with tribal group, language group, clan, mission or other Aboriginal or Torres Strait Islander regional group		No	Reference	0.226	Reference	0.798
		Yes	0.93 (0.83–1.05)		1.01 (0.91–1.14)	
Participated in Aboriginal or Torres Strait Islander cultural events or traditional activities in last 12 months	Ceremonies	No	Reference	0.847	Reference	0.241
		Yes	1.01 (0.87–1.18)		1.09 (0.95–1.25)	
	Funerals / sorry business	No	Reference	0.089	Reference	0.951
		Yes	0.89 (0.78–1.02)		1.00 (0.88–1.15)	
	NAIDOC week activities	No	Reference	0.200	Reference	0.902
		Yes	1.09 (0.96–1.23)		0.99 (0.88–1.12)	
	Sports carnivals (excluding NAIDOC week activities)	No	Reference	0.128	Reference	**0.037**
		Yes	1.1 (0.98–1.24)		1.16 (1.02–1.31)	
	Festivals or carnivals involving arts, craft, music or dance (excluding NAIDOC week activities)	No	Reference	0.483	Reference	**0.032**
		Yes	0.95 (0.81–1.11)		0.83 (0.69–1.00)	
	Involved with Aboriginal and / or Torres Strait Islander organisations	No	Reference	0.096	Reference	0.873
		Yes	1.15 (0.99–1.33)		0.98 (0.81–1.20)	
	Fished/Hunted/Gathered wild plants or berries	No		**0.003**		0.161
		Yes	0.84 (0.75–0.94)		0.90 (0.78–1.04)	
	Made Aboriginal or Torres Strait Islander arts or crafts/Performed music, dance or theatre	No	Reference	**0.019**	Reference	0.176
		Yes	1.16 (1.04–1.31)		1.10 (0.97–1.26)	
	Written or told any Aboriginal and / or Torres Strait Islander stories	No	Reference	0.392	Reference	0.863
		Yes	1.09 (0.9–1.33)		1.02 (0.85–1.21)	
	Any cultural or traditional activity	No	Reference	0.558	Reference	0.515
		Yes	0.96 (0.84–1.1)		1.07 (0.87–1.32)	

*Note*: Values significant at the 5% level are indicated in bold.

^a^ Adjusted for age, sex, work or study, remoteness areas, socioeconomic status, and all covariates presented in the table.

## 4 DISCUSSION

Reducing tobacco initiation among young people is critical for reducing the lifelong impacts associated with smoking and promoting optimal health outcomes. Recent evidence suggests there has been a reduction in the number of Aboriginal and Torres Strait Islander adolescents who smoke, and an increase in the age at which they initiate smoking.^10^ This is supported by the findings from our study, where the majority (66%) of Aboriginal and Torres Strait Islander young people aged 15–24 years of age do not smoke. Most 15–16‐year‐olds (81%) and 17–18‐year‐olds (78%) did not smoke; however, this rate declined to 57% non‐smoking by ages 19–20 years.

Living in a rural or remote location and having lower socioeconomic advantage are often considered factors associated with smoking when considered on their own.^32^, ^33^ While our findings support this, the association was no longer significant when considering other factors. This is possibly due to the influence of the work or study factor, which may play a bigger role in influencing smoking behaviour at an individual level than region alone. Previous research has observed trends between positive exposures to social determinants of health, such as greater socioeconomic position and higher educational attainment and not smoking in Aboriginal and Torres strait Islander adults.^32^, ^34^ Being occupied in employment or education may offer a protective factor against smoking through increased income, positive peer influences, reduced boredom, reduced stress, or increased exposure to health messages.^7^, ^35^ Higher levels of education are often associated with better health outcomes, higher incomes, and increased employment opportunities.^36^, ^37^ However, Aboriginal and Torres Strait Islander Australians continue to face significant systemic barriers to accessing education and employment, including the ongoing impacts of segregation and colonisation, limited opportunities, and increased rates of suspension and expulsion from school.^6^, ^38^, ^39^ It has been suggested that social determinants such as socioeconomic status and educational attainment are responsible for 34% of the health gap between Indigenous and non‐Indigenous Australians.^37^ These findings reinforce the potential impact strategies that address barriers to education and employment could have on promoting non‐smoking as well as health equity and improving wellbeing more broadly.

The role of positive peer models and strong family non‐smoking attitudes have previously been found to have a positive influence on Aboriginal and Torres Strait Islander adolescent smoking behaviour.^6^, ^7^, ^11^ This is highlighted in the current study where those living where there is no smoking inside the dwelling where more likely to not smoke. Peers and family support can also influence the use of other drugs and alcohol.^40^ In this study, we found that those who had lower illicit drug and alcohol use were also less likely to smoke. These findings suggest the need to address smoking within a social context, considering households, peers and other risk behaviours.

Cultural identity can have a positive impact on the health and wellbeing outcomes of Aboriginal and Torres Strait Islander people.^19^, ^41^ However, there is a risk of smoking becoming normalised and for smoking together to be seen as promoting connectedness in communities.^6^, ^35^, ^42^ In social settings and through cultural practices such as the exchanging and sharing of tobacco, smoking can become entrenched, making quitting or not starting difficult.^43^ In addition, Aboriginal and Torres Strait Islander smokers are less likely to view society as disapproving of smoking than smokers in the broader Australian population.^44^ In the current study, cultural factors were largely found to have no association with not smoking when accounting for other factors. Furthermore, while those who did not experience being removed from family had a higher rate of not smoking than those who did, this was not significant in regressions. It is likely that the questions asked in the ABS NATSISS Survey^24^ do not fully capture the protective effects of culture. Studies such as Mayi Kuwayu, a national longitudinal study of Aboriginal and Torres Strait Islander culture, health and wellbeing, are addressing this by identifying key cultural domains and developing a survey tool.^45^ Encouragingly, we did find that those participating in cultural sporting events were more likely to not smoke than those who do not participate. Sporting events provide opportunities for community members to come together and celebrate their culture, while also engaging in physical activity and promoting healthy lifestyles.^46^ For these reasons, sport has historically been used to address a range of issues. An example is the community‐led Aboriginal Knockout Health Challenge promoted by NSW Rugby league, which was shown to be effective in reducing weight among participants.^47^ This model could perhaps be replicated in other contexts to further promote healthy lifestyles. Given what is known about the positive impact of culture and connection on health and wellbeing outcomes it is important that culture is centred in interventions addressing smoking. Research has shown that family and kin relations are important in determining smoking behaviours among Aboriginal and Torres Strait Islander people, and that identity, empowerment, autonomy, and family and community support are key factors influencing smoking cessation.^35^, ^43^, ^48^, ^49^ Further research exploring the nuanced interactions between cultural identity and smoking behaviours is necessary to better understand these complex dynamics and to design interventions that effectively integrate cultural contexts.

A strength of the study is that it uses a nationally representative sample and takes a strength‐based approach by considering the factors associated with not smoking. This approach helps to identify protective factors that can be built upon when developing policies and initiatives to address smoking and improve health outcomes. However, care should be taken not to infer causality due to the cross‐sectional nature of the study. The survey relies on self‐reported data collected during personal interviews to assess smoking status and could therefore underestimate the true smoking rate. While another adult may have been present for the interviews, participants were able to complete the substance use form using the interviewer's laptop without the need to respond out loud in an effort to reduce this bias.^24^

## 5 CONCLUSIONS

Despite historical challenges, recent evidence suggests a positive trend in decreasing smoking prevalence among Aboriginal and Torres Strait Islander adolescents. This study used a nationally representative sample and a strength‐based approach to identify protective factors that can inform policies and initiatives to reduce smoking and improve health outcomes. Our results emphasised the need for multifaceted approaches to encourage Aboriginal and Torres Strait Islander young people to be smoke‐free and the importance of social determinants to young people's health and wellbeing. Increasing participation in education and employment through the removal of systemic and structural barriers, addressing smoking within households, and considering smoking in the context of other risk behaviours, such as drug and alcohol use, are likely to be important considerations in increasing non‐smoking among Aboriginal and Torres Strait Islander young people. While cultural identity remains a crucial factor influencing the health outcomes of Aboriginal and Torres Strait Islander people broadly, this study reveals complex associations with smoking behaviour.

## FUNDING INFORMATION

This study was funded by an NHMRC Centre for Research Excellence Grant (G1801026). Infrastructure support was provided by the HMRI Equity in Health and Wellbeing Research Program.

## CONFLICT OF INTEREST STATEMENT

The authors declare no conflicts of interest.

## Supporting information


**Data S1.** Explanation of each variable used in the analysis.

## Data Availability

The data that support the findings of this study are available on request from the corresponding author. The data are not publicly available due to privacy or ethical restrictions.
