# Supplementary material for: Factors associated with not smoking among Aboriginal and Torres Strait Islander adolescents and young people: Analysis of data from the 2014 to 15 National Aboriginal and Torres Strait Islander Social Survey
Source: Health Promot J Austr. 2024 Dec 26;36(1):e942. doi: 10.1002/hpja.942 (PMC11671702; doi:10.1002/hpja.942)
Supplement: Supplementary file 1 — Data S1. Explanation of each variable used in the analysis. [file HPJA-36-0-s001.docx]

**Supplementary file:** Explanation of each variable used in the analysis.

| **Variable** | **NATSIS Question ID** | **Notes for analysis** |
| --- | --- | --- |
| **Socio-economic status** |  | Presented as quintiles calculated from home postcode using the 2011 Socio-Economic Index for Areas (SEIFA) Index of disadvantage^a^. |
| **Geographical remoteness** |  | Classified from home postcode using the 2011 Australian Statistical Geography Standard classifications Accessibility/Remoteness Index of Australia Plus (2011 ARIA+) score^b^.   - Remote or very remote (remote) - Major city, inner regional or outer regional (Non-remote) |
| **Indigenous status** | **ATSI_Q03** | Recorded as Aboriginal, Torres Strait Islander or Both Aboriginal and Torres Strait Islander |
| **Age** | CDOB_Q01 | Provided as age in years from 15 to 24. and categorised into 2 year intervals |
| **Sex** | D2_Q06 | Recorded as Male or Female |
| **Work or Study** | Q01EP  Q01WD | Accessed as combined work/study variable   - Working full-time (Employed) - studying full-time (studying) - Working part-time and studying part time (Both studying and employed) - Working part-time only (Employed) - Studying part-time only (Studying) - Not working or studying (not employed or studying) |
| **Current smoking status** | Q01SMO | Do you currently smoke?   - Yes (Smoker) - No (non-smoker) |
| **Type of smoker** | Q02SMO | Do you smoke regularly, that is, at least once a day?   - Yes (daily smoker) - No (occasional smoker) |
| **Type of non-smoker** | Q05SMO | Have you ever smoked regularly?   - Yes (ex-smoker) - No (never smoker) |
| **Self-assessed health status** | Q01STAT | In general, would you say your health is  Excellent  very good  good  fair  poor |
| **Psychological distress (Kessler 5)** | SEWBQ01-SEWBQ05 | This is a modified version of the 5 item Kessler Psychological Distress Scale (K5)^c^ with some slight wording variations.  Distress score categories:   - 5–7.99 (low) - 8–11.99 (moderate) - 12–14.99 (high) - 15– 25 (very high) - Unable to determine (low) |
| **Overall life satisfaction** | OLS_Q01 | Overall, how satisfied are you with life as a whole these days?  Rating of 0 (not at all satisfied) to 10 (completely satisfied) |
| **Any mental health conditions** | LTC_Q02 | Have you ever been told by a doctor or nurse that you have any of these conditions?  depression or feeling depressed (yes)  Anxiety or feeling anxious or nervous(yes)  behavioural or emotional problems (yes) |
| **Alcohol consumption- lifetime (long term) risk** | Q02ALC  Q03ALC | Calculated by ABS from 2 questions^d^:  How often do you drink (alcohol/grog)?  and  What do you usually drink in a day? (Type of drink, volume and quantity recorded)   - more than 2 standard drinks per day on average last week (yes) - less than 2 standard drinks per day on average last week (no) - Not stated (no) |
| **Alcohol consumption- single occasion (short term) risk** | Q03ALC | Thinking about days that you drink, what do you usually drink in a day?   - 5+ drinks consumed (yes) - <5 drinks consumed (no) - Last consumed alcohol 12 months or more ago (no) - Never consumed alcohol (no) - Not stated (no) |
| **Number of different types of substances used in last 12 months** | Module 21.01 (Remote) and Module 21.02 (non-remote) | In the last 12 months have you used?:  -Pain killers or analgesics for non-medical purposes  -Tranquillisers of sleeping pills for non-medical purposes  -Marijuana, Hashish or Cannabis resin  -Methadone  -sniffed petrol  -other inhalants  -Kava  -Amphetamines or speed  - Heroin  -Cocaine  -LSD or Synthetic Hallucinogens  -Naturally occurring Hallucinogens  -Ecstacy or designer drugs  -Other substances like these  All yes responses summed to give total number of different substances used |
| **Smoke free inside house** | Q02HHS | Does anybody ever smoke inside this house/dwelling?   - Yes - No - Not stated (no) |
| **Whether ever removed from natural family** | Q01FMTK | Have you been removed from your family by welfare or the government or taken away to a mission?   - Yes (Has been removed from natural family) - No (Has never been removed from natural family) - Don’t want to talk about it (Not answered) |
| **Whether relatives ever removed from natural family** | Q02FMTK | Have any of your relatives been removed from their family by welfare or the government or taken away to a mission?   - Yes (Relatives removed from natural family) - No (Relatives never removed from natural family) - Don’t know (Not answered) - Don’t want to talk about it (Not answered) |
| **Speak any Aboriginal or Torres Strait Islander language** | Q02LANG | Do you speak any Aboriginal or Torres strait Islander languages   - Yes - Yes some words only - No |
| **Currently lives in homelands / traditional country** | Q02CULI  Q03CULI | Combined from two questions:  Do you recognise an area as your homelands or traditional country?   - Yes - No   and  Do you currently live there?   - Yes - No |
| **Identify with clan, tribal or language group** | Q01CULI | Dichotomised response to question “Do you identify with any of these?”   - a tribal group (yes) - a language group (yes) - a clan (yes) - a mission (yes) - Aboriginal or Torres Strait Islander regional group (yes) - None of the above (no) |
| **Participation in Aboriginal or Torres Strait Islander cultural or traditional activities in last 12 months** | Q01CULP  Q03CULP | List of activities and ceremonies combined from two questions:  In the last 12 months have you gone to or been involved in any of these Aboriginal or Torres Strait Islander cultural activities or ceremonies? (Yes/No for each)   - Ceremonies - Funerals / sorry business - NAIDOC week activities - Sports carnivals (excluding NAIDOC week activities) - Festivals or carnivals involving arts, craft, music or dance (excluding NAIDOC week activities) - Been involved with Aboriginal or Torres Strait Islander organisations - None of the above   and  Have you done any of the following activities in the last 12 months? (Yes/No for each)   - Fished/Hunted/Gathered wild plants/berries (combined) - Made Aboriginal or Torres Strait Islander arts or crafts/Performed music, dance or theatre (combined - Written or told any Aboriginal and / or Torres Strait Islander stories - None of the above |

a. Technical Paper Socio-Economic Indexes for Areas (SEIFA), 2011. Cat no. 2033.0.55.001 (2011).

b. Australian Statistical Geography Standard (ASGS): Volume 1 - Main Structure and Greater Capital City Statistical Areas (2011).

c. McNamara BJ, Banks E, Gubhaju L, et al. Measuring psychological distress in older Aboriginal and Torres Strait Islanders Australians: a comparison of the K‐10 and K‐5. Australian and New Zealand journal of public health. 2014;38(6):567-573.

d. Australian Bureau of Statistics. National Aboriginal and Torres Strait Islander Health Survey methodology: 2019.

https://www.abs.gov.au/methodologies/national-aboriginal-and-torres-strait-islander-health-survey-methodology/2018-19
